# Complete genomic sequence of a novel phytopathogenic *Burkholderia* phage isolated from fallen leaf compost

**DOI:** 10.1007/s00705-020-04811-3

**Published:** 2020-10-30

**Authors:** Ryota Sasaki, Shuhei Miyashita, Sugihiro Ando, Kumiko Ito, Toshiyuki Fukuhara, Richard Kormelink, Hideki Takahashi

**Affiliations:** 1 Graduate School of Agricultural Science, Tohoku University, Sendai, Miyagi 980-0845 Japan; 2 Department of Applied Biological Sciences and Institute of Global Innovation Research, Tokyo University of Agriculture and Technology, Fuchu, Tokyo 183-8538 Japan; 3 Laboratory of Virology, Wageningen University, 6708 PB Wageningen, The Netherlands

## Abstract

In contrast to most *Burkholderia* species, which affect humans or animals, *Burkholderia glumae* is a bacterial pathogen of plants that causes panicle blight disease in rice seedlings, resulting in serious damage to rice cultivation. Attempts to combat this disease would benefit from research involving a phage known to attack this type of bacterium. Some *Burkholderia* phages have been isolated from soil or bacterial species in the order *Burkholderiales*, but so far there has been no report of a complete genome nucleotide sequence of a phage of *B. glumae*. In this study, a novel phage, FLC5, of the phytopathogen *B. glumae* was isolated from leaf compost, and its complete genome nucleotide sequence was determined. The genome consists of a 32,090-bp circular DNA element and exhibits a phylogenetic relationship to members of the genus *Peduovirus*, with closest similarity to *B. multivorans* phage KS14. In addition to *B. glumae,* FLC5 was also able to lyse *B. plantarii,* a pathogen causing rice bacterial damping-off disease. This is the first report of isolation of a P2-like phage from phytopathogenic *Burkholderia*, determination of its complete genomic sequence, and the finding of its potential to infect two *Burkholderia* species: *B. glumae* and *B. plantarii*.

**Electronic supplementary material:**

The online version of this article (10.1007/s00705-020-04811-3) contains supplementary material, which is available to authorized users.

*Burkholderia* is the type genus of family *Burkholderiaceae*, which, together with a number of ecologically diverse organisms, comprises the order *Burkholderiales*, class *Betaproteobacteria*. The order *Burkholderiales* includes 10 other genera in addition to *Burkholderia* [1] and includes truly environmental saprophytic organisms, phytopathogens, and opportunistic pathogens that infect humans and animals. Bacteriophages are also widespread in the biosphere and probably have an important influence on the evolution, diversity, and ecology of environmental bacteria [2–4].

So far, 37 complete genome sequences of *Burkholderia* phages have been registered in the NCBI genome database [1, 5–8], isolated from *Burkholderia cepacia* (an opportunistic human pathogen that most often causes pneumonia), *B. cenocepacia* (another opportunistic pathogen causing infections in patients with cystic fibrosis and chronic granulomatous disease), *B. pseudomallei* (causing melioidosis), and *B. thailandensis* (closely related to *B. pseudomallei*). Also, related prophage islands, diversified and modulated, have been identified in *Burkholderia* genomes [9–12].

Concerning the phytopathogenic species *B. glumae*, which causes panicle blight and seedling rot diseases, thereby reducing rice yield [13, 14], the whole genome sequences of seven strains of *B. glumae* (BGR1, PG1, LMG2196, AU6208, 3252-8, 336gr-1, and NCPPB3923) have been registered in the NCBI genome database [15] and shown to contain *B. glumae* prophage DNA [16, 17]. In addition, *B. glumae* phages have been isolated from river or puddle water [18]. However, to date, there is no complete genome DNA sequence of a *B. glumae* phage. Therefore, efforts were made to isolate *B. glumae* phages from leaf compost. This is the first report of the complete genome nucleotide sequence of a P2-like phage that lyses two plant-pathogenic *Burkholderia* species: *B. glumae* and *B. plantarii*.

The phage particle isolation procedure is described in the Electronic Supplementary Materials. Electron microscopy of the phage particle showed that it consists of an icosahedral capsid with a contractile tail (Fig. S1B). Tailed phages belong to the families *Myoviridae*, *Podoviridae* or *Siphoviridae* in the order *Caudovirales*, contain double-stranded DNA (dsDNA), and represent the most numerous, the most widely distributed, and probably the oldest group of bacteriophages [19, 20].

Isolation of phage DNA, construction of the phage DNA library, DNA sequencing and data analysis are described in the Electronic Supplementary Materials. The genome of the *Burkholderia* phage consisted of a 32,090-bp circular dsDNA molecule (Fig. 1A). The restriction enzyme digestion patterns obtained after cutting the circular genomic DNA with *Afl* II or *Nar* I were in agreement with the restriction map predicted from the nucleotide sequence (Fig. 1A and B). A cohesive end site required for termination of packaging of the phage chromosome is labeled as “cos” in Fig. 1A. Detection of 1.7- and 1.8-kbp DNA fragments, which seem to be generated from a 3.5-kbp DNA fragment by treatment at 80 °C for 15 min (Fig. 1C), and alignment of core nucleotide sequence including cos (Fig. S2) suggested that the cos sequence is present in the genome of the *Burkholderia* phage. The genome nucleotide sequence has been submitted to the GenBank/EMBL/DDBJ database with accession number LC528882, in which the terminal nucleotide of the cos site is oriented as + 1 (Fig. S2).

**Fig. 1:**
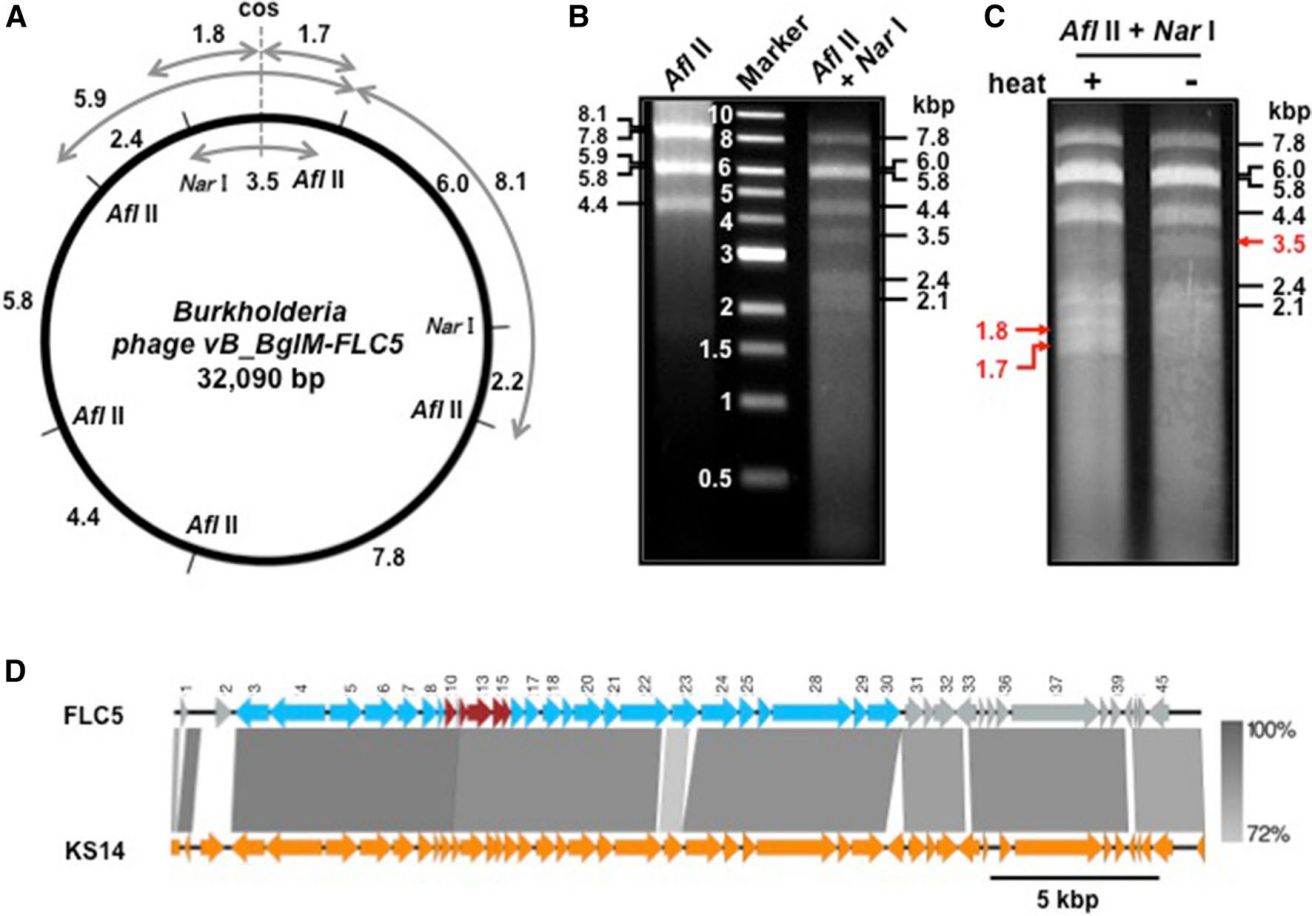
Genomic structure of *Burkholderia phage vB_BglPM-FLC5*. **A**. Schematic representation of the genomic DNA, showing cleavage sites for *Afl*II and *Nar*I. Numbers represent the size of fragments in nucleotide base pairs (kbp) created by digestion with *Afl*II and/or *Nar*I. **B**. Electrophoretic analysis of genomic DNA of FLC5 digested with *Afl*II (left lane) or *Afl*II and *Nar*I (right lane). Center lane: DNA ladder size marker. **C.** Electrophoretic analysis of genomic DNA of FLC5 digested with *Afl*II and *Nar*I, followed by heating at 80 °C for 15 min (heat +) and then chilling on ice for 5 min. As a control, *Afl*II and *Nar*I-digested FLC5 DNA was not treated at 80 °C (heat -). A cohesive end site required for termination of packaging of the phage chromosome is labeled as “cos”. 1.7- and 1.8-kbp DNA fragments, which appear to have been generated from an unheated 3.5-kbp DNA fragment containing “cos” by heat treatment, are indicated by red arrows. **D**. Functional gene map and comparative genomic analysis of *Burkholderia* phage FLC5 of *B. glumae* and KS14 of *B. multivorans*. Red arrows represent the host lysis module; light blue arrows represent the phage structure and packaging module; and gray arrows represent genes encoding the DNA partitioning protein, zinc finger CHC2-family transcription factor, repressor protein, serine recombinase and hypothetical proteins

Nucleotide sequence comparisons, using BLASTn revealed 92% identity (query coverage, 89%) to the complete DNA genome sequence of *Burkholderia* phage KS14, which was isolated from an extract of *Dracaena* sp. soil plated on *B. multivorans* C5393 [21] and belongs to the family *Myoviridae,* subfamily *Peduovirinae*, genus *Peduovirus*. Thus, the purified phage was assigned the name "Burkholderia phage vB_BglM-FLC5", derived from ʻa **v**irus of **B**acteria, infecting ***Burkholderia glumae***, with ***Myoviridae ***morphologyʼ, and the strain was designated "FLC5".

Open reading frames (ORFs) encoding gene products gp1 to 45 were identified and assigned to the FLC5 genome DNA by the method described in the Electronic Supplementary Materials (Fig. 1D and Table S1). The functions of proteins encoded by the FLC5 genome were predicted by homology searches using BLASTp. ORFs 10 to 15 encode a host lysis module, and a phage structure and packaging module is encoded by ORFs 3–9 and 16–30, respectively (Fig. 1D and Table S1). Serine recombinase, repressor protein, zinc finger CHC2-family transcription factor, and a DNA partitioning protein are encoded by ORFs 33, 34, 38 and 45, respectively (Fig. 1D and Table S1).

A multiple sequence alignment was performed of the genome sequence of *Burkholderia* phage FLC5 and those of 16 other *Myoviridae* members*.* The latter included 11 *Peduovirus* phages and four *Hpunavirus* phages, forming the in-group compared against *Muvirus* phage Mu of *Escherichia*. The analysis revealed the highest sequence identity of FLC5 to phage KS14 of *B. multivorans* (i.e., *Burkholderia* phage KS14) and, accordingly, the closest relationship in the phylogenetic tree (Fig. 2). Considering that *Burkholderia* phage KS14, which is the closest relative of FLC5, is a temperate phage [22], FLC5 may not be obligately lytic but temperate. It is also possible that FLC5 may be a mutant lacking ability to lysogenize.

**Fig. 2:**
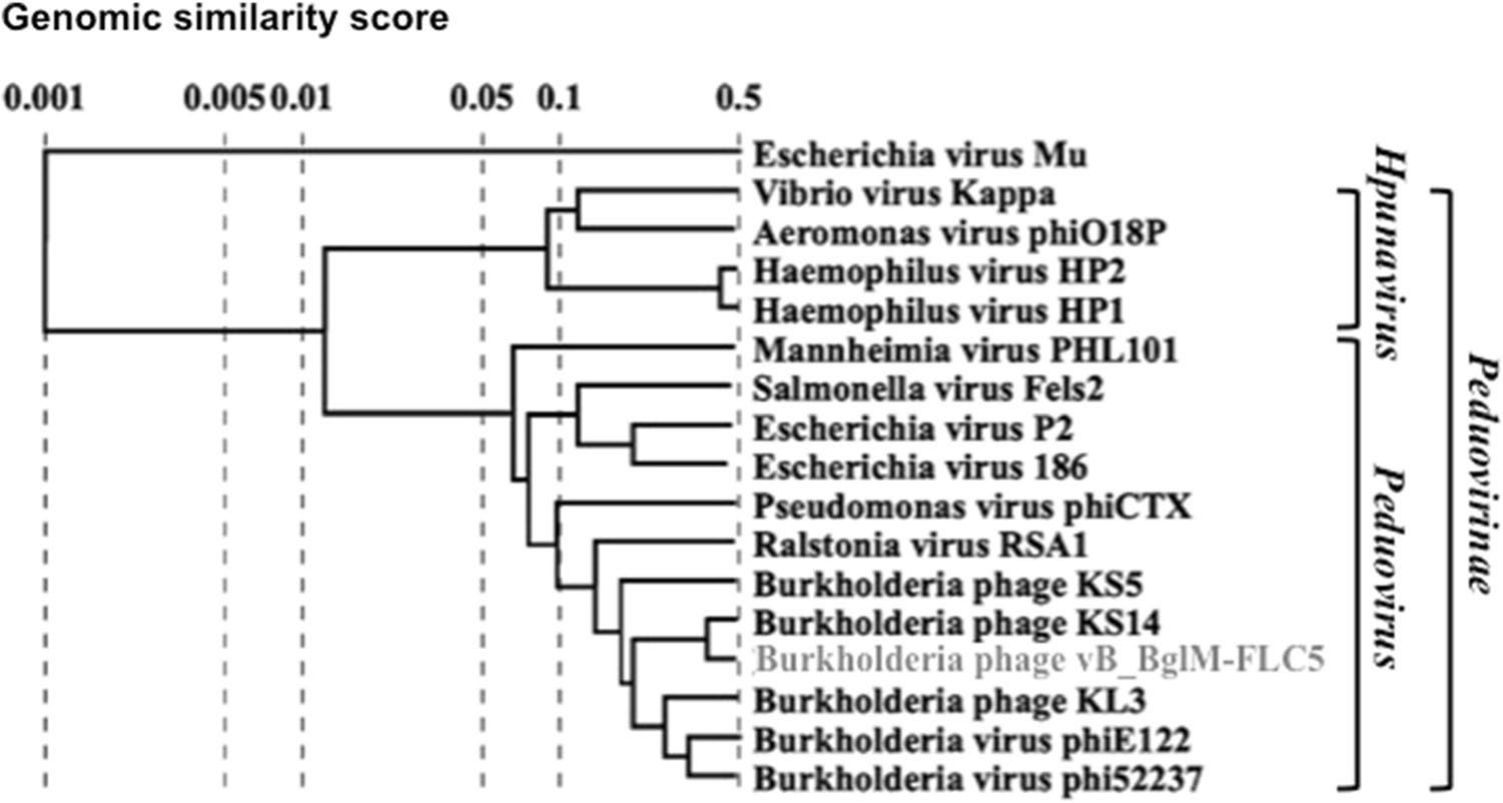
Whole-genome phylogenetic tree of *Burkholderia* phage FLC5 and 15 members of the subfamily *Peduovirinae*, including 11 members of the genus *Peduovirus*, four members of the genus *Hpunavirus*, and one member of the genus *Muvirus* (phage Mu of *Escherichia*) generated using ViPTree v1.9 [8]

Upon PCR analysis of genomic DNA from *B. glumae* MAFF302746 using primers specific for phage FLC5 (see Methodology in Electronic Supplementary Materials), no amplified DNA products were obtained, whereas the 16S rDNA fragment of *B. glumae* MAFF302746, which was used for isolation of FLC5 in our experiment, was detected by PCR using primers specific for 16S rDNA (Fig. S3). This indicated that FLC5 was not derived from prophage sequences in the *B. glumae* MAFF302746 genome but instead from a *Burkholderia* phage collected from leaf compost.

Rice panicle blight and seedling rot caused by *B. glumae* and rice bacterial damping-off and seedling blight caused by *B. plantarii* are major bacterial diseases affecting rice nursery seedling cultivation [13, 14, 23]. To survey the host range of *Burkholderia* phage FLC5, the susceptibility of five isolates of *B. glumae* and six isolates of *B. plantarii* was examined. Three isolates of *B. glumae* (MAFF302746, MAFF301169 and MAFF302417) and one isolate of *B. plantarii* (MAFF302475) were lysed by *Burkholderia* phage FLC5 (Table S2), demonstrating that it has the potential to control two major bacterial diseases of rice nursery seedling cultivation.

**Nucleotide sequence accession number** The GenBank/EMBL/DDBJ accession number for Burkholderia phage vB_BglM-FLC5 is LC528882.

## Electronic supplementary material

Below is the link to the electronic supplementary material.

Supplementary file1 (DOCX 2476 kb)

Supplementary file2 (XLSX 13 kb)

Supplementary file3 (TXT 62 kb)
