# Supplementary material for: Complete genomic sequence of a novel phytopathogenic Burkholderia phage isolated from fallen leaf compost
Source: Arch Virol. 2020 Oct 30;166(1):313–6. doi: 10.1007/s00705-020-04811-3 (PMC7815583; doi:10.1007/s00705-020-04811-3)
Supplement: Supplementary file 1 — Supplementary file1 (DOCX 2476 kb) [file 705_2020_4811_MOESM1_ESM.docx]

**Electronic supplementary materials**

**Materials and Methods**

*Isolation of phage particles from fallen leaf compost*

Fallen leaf compost was prepared by layering 135 L fallen hardwood leaves, 45 L rice bran and 20 L water in a heap and then mixing the layers together after the temperature of the heap increased to more than 60°C. The mixing procedure was repeated five times to maintain aerobic status for maturation of the compost. A 6.5 g sample (fresh weight) of fallen leaf compost was added to 25 mL potato-peptone-glucose (PPG) liquid medium and shaken vigorously at 25°C. At 2 h after shaking, the suspension was centrifuged at 5000 ×*g* for 5 min at 25°C. The supernatant was filtered through a nitrocellulose membrane filter (0.45 μm pore size) and added to 2 ml of 1 × 10^7^ cells/mL *Burkholderia glumae* MAFF302746, which was kindly supplied by the NARO Genebank (Tsukuba, Japan). After incubation for 20 h at 25°C, the enriched culture was centrifuged at 5000 ×*g* for 5 min at 25°C. A 0.5 mL sample of supernatant was again filtered through a 0.45 μm nitrocellulose membrane filter and added to 0.5 ml of 1 × 10^7^ cells/mL of *B. glumae* MAFF302746. After incubation for 10 min at 25°C, the filtrate was added to 5 mL PPG top agar medium containing 0.5% agar and immediately poured onto a PPG 1.5% agar medium plate according to the standard protocol [1].

After incubation overnight at 25°C, plaques were observed on the plate (Fig. S1A). A plaque was picked up with surrounding bacteria and transferred to PPG liquid medium. After culturing overnight at 25°C with 180 rpm rotation, phage particles were purified from bacterial lysates using NaCl and PEG8000 according to the standard method [2].

*Isolation of phage DNA, construction of phage DNA library, DNA sequencing, and data analysis.*

Genomic DNA was extracted from purified phage particles using the standard procedure [1]. A phage DNA library was constructed using the NexteraXT DNA Library Prep Kit (Illumina, San Diego, CA) and sequenced on the Illumina MiSeq according to the instruction manual. The complete FLC5 genomic sequence was assembled using SPAdes genome assembler v3.13.0 [3].

Open reading frames (ORFs) encoding gene products 1 through 45 were assigned on the FLC5 genome using GeneMark.hmm with heuristic parameters [4] with modified gene prediction of three ORFs encoding gp10, gp19, and gp31 according to the corresponding ORFs of Peduovirus [5, 6].

Comparative genomic analysis was performed and the functional gene map of *Burkholderia* phages FLC5 and KS14 shown in Fig. 1C was constructed using EasyFig v2.2.2 [7]. The whole-genome phylogenetic tree of phage FLC5 of *Burkholderi*a and other phages in Fig. 2 was constructed using ViPTree v1.9 [8].

*Detection of phage FLC5 genome fragment by PCR*

PCR was conducted at 94°C for 30 s, 60°C for 45 s and 72°C for 2 min with 30 cycles using the FLC5-specific primers FLC5-F1 (5′-CAGGTTGAACGCCTTAAGTTTC-3′) and FLC5-R1 (5′-GGCCGAGGATCACTACAACTAT-3′) with genomic DNA of *B. glumae* MAFF302746 as a template. *B. glumae* MAFF302746 had been used for propagation of FLC5. To confirm the input of *B. glumae* MAFF302746 as the template for PCR, PCR was done using 16S rDNA-specific primers 63f (5′-CAGGCCT- AACACATGCAAGTC-3′) and 1378r (5′-CGGTGTGTACAAGGCCCGGGAACG-3′) with same genomic DNA at same condition described above.

**References**

1. Sambrook J, Russell DW (2001) Extraction of bacteriophage λ DNA from large-scale cultures using proteinase K and SDS. In: Sambrook J, Russell DW (eds) Molecular cloning: a laboratory manual, 3rd edn. Cold Spring Harbor Laboratory Press, New York, pp2.56-2.58

2. Yamamoto KR, Alberts BM, Benzinger R, Lawhorne L, Treiber G (1970) Rapid bacteriophage sedimentation in the presence of polyethylene glycol and its application to large-scale virus purification. Virology 40:734-744

3. Nurk S, Bankevich A et al. (2013) Assembling genomes and mini-metagenomes from highly chimeric reads. In: Deng M., Jiang R., Sun F., Zhang X. (eds) Research in Computational Molecular Biology. RECOMB 2013. Lecture Notes in Computer Science, 7821, pp158-170, Springer, Berlin, Heidelberg.

4. Besemer J, Borodovsky, M (1999) Heuristic approach to deriving models for gene finding. Nucl Acid Res 27:3911-3920

5. Julien B, Lefevre P, Calendar R (1997) The two P2 Ogr-like domains of the Delta protein from bacteriophage P4 are required for activity. Virology 230:292-299

6. Lynch, K.H., Stothard, P. Dennis, J.J. (2010) Genomic analysis and relatedness of P2-like phages of the *Burkholderia cepacia* complex. BMC Genomics 11:599

7. Sullivan MJ, Petty NK, Beatson SA (2011) Easyfig: a genome comparison visualizer. Bioinformatics 27:1009-1010

8. Nishimura Y, Yoshida T, Kuronishi M, Uehara H, Ogata H, Goto S (2017) ViPTree: the viral proteomic tree server. Bioinformatics 33:2379-2380

**Fig. S1** Phage plaques of phage FLC5 of *Burkholderia* and transmission electron microscopic image of the FLC5 phage particle. **A**. Phage plaques of *Burkholderia* phage FLC5 on a Petri dish of cultured *Burkholderia glumae* MAFF302746. **B**. Transmission electron microscopic image of the FLC5 phage particle with an icosahedral capsid with contractile tail.

**
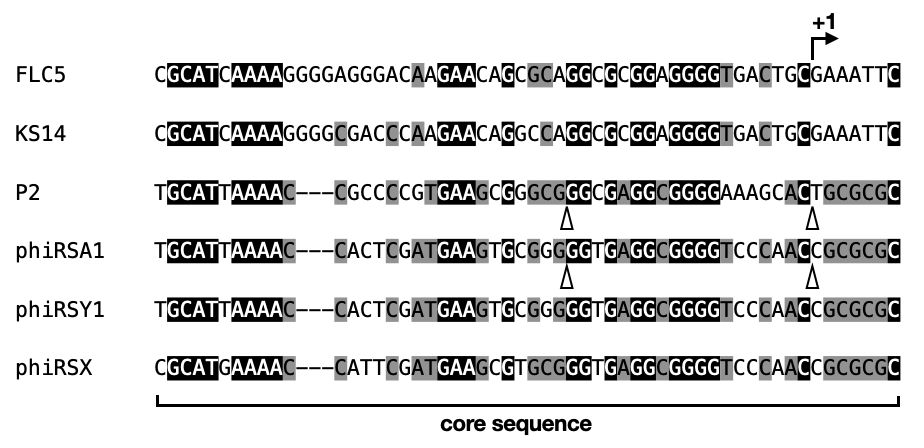
**

**Fig. S2** Alignment of core nucleotide sequence including cos (cohesive end site) site of Burkholderia phage FLC5 and five other common peduoviruses. The terminal nucleotide of the cos site is oriented as +1. The triangles indicate the cleavage sites. Nucleotides with a black background indicate identical core sequences for all six phages; those with a gray background was four and five core sequences of six phages.

**10**

**8**

**6**

**5**

**4**

**3**

**2**

**1.5**

**1**

**0.5**

**kbp**


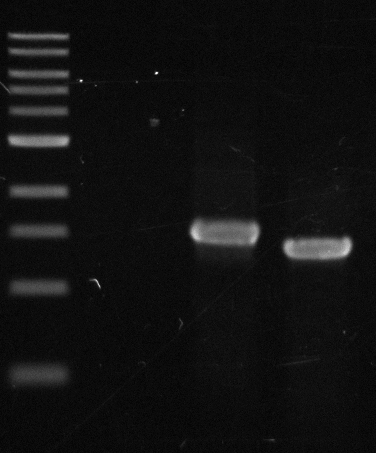


**Marker**

***B. glumae***

**MAFF302746**

**Burkholderia**

**phage FLC5**

***B. glumae***

**MAFF302746**

**FLC5-specific**

**16S-specific**

**Fig. S3** PCR products amplified from phage FLC5 of *Burkholderia*. PCR was conducted using FLC5-specific primers and genomic DNA of either *Burkholderia* phage FLC5 or *Burkholderia glumae* MAFF302746, which was used for isolation of FLC5 in our experiment, as template (FLC5-specific). *B. glumae* MAFF302746 was used to propagate FLC5. To confirm the input of *B. glumae* MAFF302746 as the template for PCR, PCR product amplified from same template DNA with 16S rDNA-specific primers, was shown as a positive control at right lane (16S-specific). Ladder DNA size marker was applied at left lane.
